# Global burden of colorectal cancer attributable to high fasting plasma glucose from 1990 to 2021 and projection to 2040

**DOI:** 10.3389/fonc.2025.1590382

**Published:** 2025-08-26

**Authors:** Yuting Li, Weiying Hong, Peiqing Chen, Yu Zhang

**Affiliations:** Department of Interventional Endoscopy, Cancer Hospital of Shantou University Medical College, Shantou, Guangdong, China

**Keywords:** colorectal cancer, high fasting plasma glucose, global burden of disease, mortality, disability-adjusted life years

## Abstract

**Background:**

High fasting plasma glucose (HFPG) is an established risk factor for colorectal cancer (CRC). This study analyzes global epidemiological patterns and temporal trends of HFPG-attributable CRC burden from 1990 to 2021, with projections to 2040.

**Methods:**

Using Global Burden of Disease 2021 data, we assessed geographical, sex-specific, age-related, and socio-demographic index (SDI) variations in mortality and disability-adjusted life years (DALYs). Joinpoint regression analysis was employed to evaluate the trends and inflection points, and the Bayesian Age-Period-Cohort (BAPC) model was performed to project future burden.

**Results:**

In 2021, HFPG accounted for 82,421 CRC deaths (95% UI: 42,427–125,402) and 1,750,923 DALYs (95% UI: 900,573–2,657,995) globally - marking substantial increases since 1990. Age-standardized mortality rates (ASMR) and DALY rates (ASDR) showed modest increases during 1990–2021 with an AAPCs of 0.31, but significant decline were identified post-2019, particularly in ASMR (APC = -0.94, *p* < 0.05). Regionally, the highest burden were noted in high SDI region, Central Europe, Barbados, and Hungary, with China contributing the absolute numbers of CRC deaths and DALYs. The burden was more pronounced among males and the elderly, with a notable emerging trend of rapidly burden increasing among young males, especially in the 30–34 age group. Projections suggest continued declines in global ASMR and ASDR through 2040.

**Conclusion:**

Despite global increases in HFPG-attributable CRC burden since 1990, recent declining trends (2019 onward) suggest potential progress in burden mitigation. Considering COVID-19 pandemic impacts on healthcare systems, post-2019 trends require cautious interpretation. Further longitudinal studies are essential to validate these trends. Given the significant variations across gender, age, and SDI, targeted interventional strategies should be developed and implemented to effectively manage this disease burden.

## 1 Introduction

Colorectal cancer (CRC) is one of the leading causes of cancer deaths and primarily develops from two distinct types of precancerous polyps including tubular adenomas and serrated polyps (1, 2). According to the GLOBCAN 2020 database, CRC ranks third in incidence and second in mortality among all cancer types, with an estimated 1.93 million new case and 0.93 million fatalities reported in 2020 (3). Therefore, CRC has been recognized as a substantial public health challenge.

Previous researches have demonstrated that the occurrence and progression of CRC are associated with numerous factors, including hereditary predisposition, dietary habits, smoking, obesity and hyperglycemia, most of which are modifiable (4–6). High fasting plasma glucose (HFPG), manifesting as prediabetes and diabetes conditions is an abnormal metabolic state (7). With the economic growth, HFPG or diabetes has become increasingly prevalent in both developed and developing countries (8, 9). As one of the leading metabolic risk factors contributing to global mortality and disability, HFPG caused approximately 5.3 million deaths and 155.7 million disability-adjusted life years (DALYs) in 2021 (10). Studies have reported that HFPG or diabetes is associated with an elevated risk of various malignant neoplasms, including CRC (11–15). Furthermore, it was reported that CRC patients with concurrent diabetes face higher risks of all-cause and cancer-specific mortality and demonstrate poorer disease-free survival, compared to non-diabetic patients (16, 17). In 2019, HFPG ranked as the sixth major risk factor for CRC globally, and particularly as the third main risk factor among female (4).

While previous study has described the global burden of CRC attributable to HFPG using GBD 2019 data, focusing primarily on regional differences, comprehensive analysis of age and sex differences in disease burden remains limited (18). In this study, we utilized updated data from GBD 2021 database to analyze trends and epidemiological features of HFPG-related CRC at global, regional, and national levels from 1990 to 2021, stratified by sex and age. Additionally, we employed the Bayesian Age-Period-Cohort (BAPC) analysis to forecast the disease burden until 2040. This study aimed to inform strategies for colorectal cancer prevention, plasma glucose control, and public health policy development.

## 2 Methods

### 2.1 Data source

The data for this study were sourced from the Global Burden of Disease (GBD) 2021 study (https://ghdx.healthdata.org/gbd-2021), which provided comprehensive assessment of 371 diseases and injuries, as well as 88 risk factors in 204 countries and territories (9, 10). The original GBD analysis applied the Comparative Risk Assessment (CRA) framework to address disease complexity through a causal web targeting hierarchically organized modifiable risks. For HFPG-attributable outcomes, this framework adjusts for key confounders such as body mass index (BMI) and dietary patterns through integrated covariate modeling and mediation analysis (10).

To ensure data robustness, GBD 2021 addressed missing data and reporting inconsistencies through integrating global data from multiple sources (e.g., scientific literature, household surveys, surveillance systems, vital registrations), applying bias adjustment via MR-BRT meta-regressions, and implementing advanced modeling (e.g., DisMod-MR 2.1, ST-GPR) to generate estimates for sparse or missing data (9). Geographically, GBD 2021 divided 204 countries and territories into 21 regions based on socioeconomic classification using the Socio-demographic index (SDI). The SDI, ranging from 0 to 1, is a composite indicator to measure the national per capita income, average education level and total fertility rate. Based on the SDI values, the countries are categorized into five groups, including high, high-middle, middle, low-middle and low SDI, with higher SDI indicating more advanced socioeconomic development.

In this study, we extracted the data on CRC burden attributable to HFPG from 1990 to 2021. In the GBD 2021 Study, HFPG was defined as fasting plasma glucose greater than 4.9–5.3mmol/L (10). The extracted metrics included the number of CRC deaths, disability-adjusted life years (DALYs), age-standardized mortality rate (ASMR), and age-standardized DALY rate (ASDR) all provided with 95% uncertainty intervals (UIs).

### 2.2 Statistical analysis

The Joinpoint regression analysis was employed to evaluate the temporal trends in ASMR and ASDR of CRC attributable to HFPG, using Joinpoint software (version 5.4.0). This method segments continuous time-series data into distinct linear trend, calculating the annual percentage change (APC) for each segment. The average annual percentage change (AAPC) a geometrically weighted average of individual APCs, was computed to assess overall trends throughout the study period (19). Both APC and AAPC were reported with 95% confidence intervals (CI). Trends were interpreted as follows: positive values in both APC or AAPC and its 95% CI indicated an upward trend, negative values suggested a downward trend, and when the 95% CI included zero, the age-standardized rate (ASR) was considered stable for that period.

To project the disease burden of CRC attributable to HFPG from 2022 to 2040, this study implemented the Bayesian Age-Period-Cohort (BAPC) analysis. The BAPC model extends the traditional generalized linear model (GLM) framework within a Bayesian context, dynamically incorporating the effects of age, period, and cohort (20). This methodology is particularly valuable in large-scale epidemiological research due to its robust handling of age-structured population data and complex cohort effects (21). Furthermore, the model’s ability to avoid parametric assumptions while maintaining predictive accuracy makes it especially suitable for disease burden forecasting (22). Using historical disease burden data from 1990 to 2021, and GBD population projection data, we employed the R packages “BAPC” and “INLA (integrated nested Laplace approximation)” to fit the model, and generate sex-specific projections through 2040 (21, 23).

Except for the Joinpoint regression analysis, all statistical analyses and visualizations in this study were conducted using R software (version 4.2.0), and statistical significance was set at *p* < 0.05.

## 3 Results

### 3.1 Global spatial distribution of colorectal cancer burden attributable to HFPG

**Figure 1:**
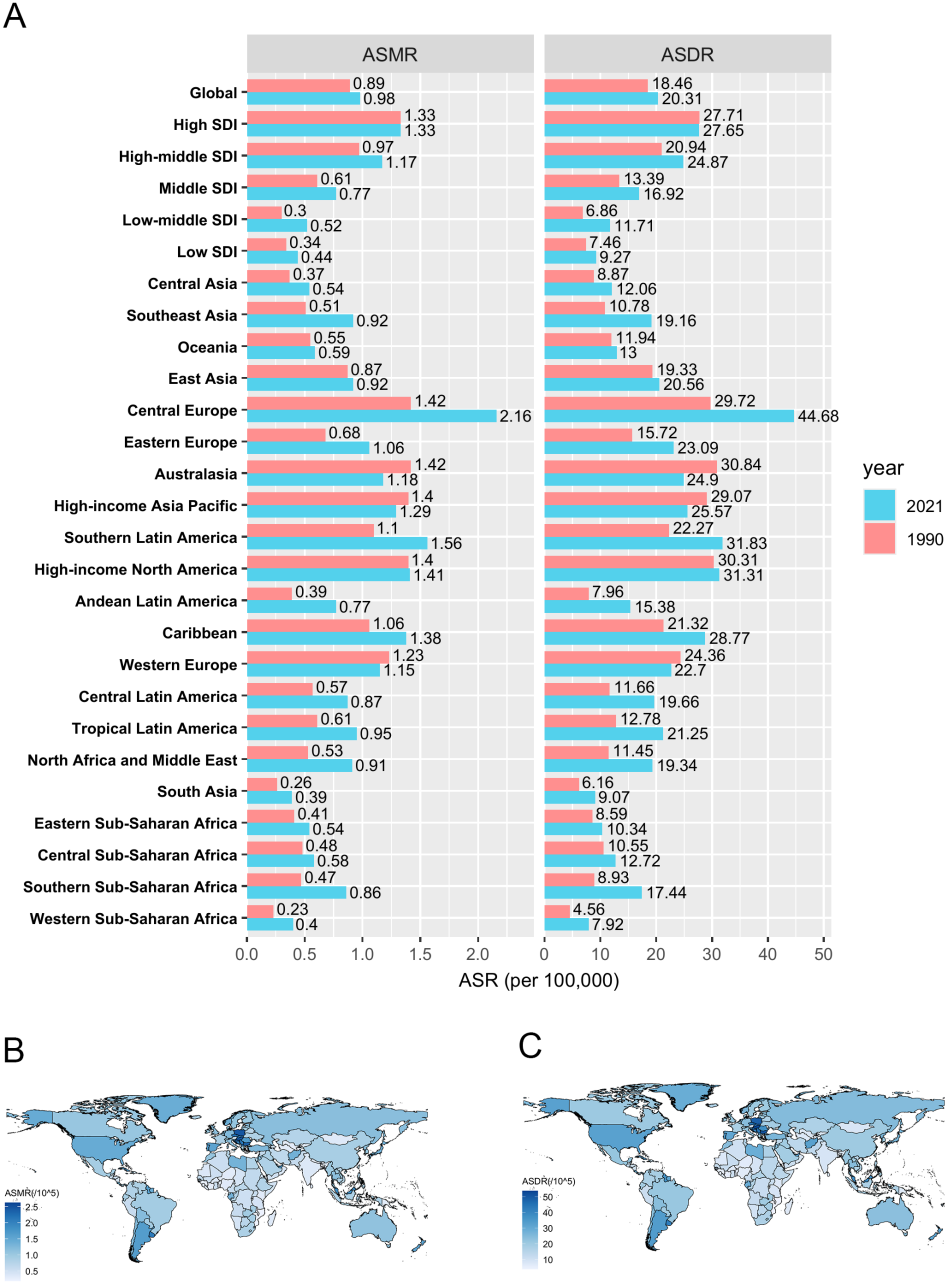
The spatial distribution of CRC burden attributable to HFPG. **(A)** The ASMR and ASDR in different regions in 1990 and 2021; **(B)** The ASMR in 204 countries and territories in 2021; **(C)** The ASDR in 204 countries and territories in 2021. ASMR, age-standardized mortality rate; ASDR, age-standardized disability-adjusted life-year rate. DALYs, disability-adjusted life-years.

Globally, in 2021, the number of CRC deaths and DALYs attributable to HFPG were approximately 82,421 (95% UI: 42,427–125,402) and 1,750,923 (95% UI: 900,573–2,657,995), respectively, representing 1.58-fold and 1.45-fold increases compared to 1990. The male-to-female ratio increased to 1.30 for deaths and 1.43 for DALYs. The ASMR and ASDR of HFPG-related CRC were 0.98 (95% UI: 0.51–1.49) and 20.31 (95% UI: 10.46–30.81) per 100,000 population (**Figure 1A**, **Tables 1**, **2**).

**Table 1:** Deaths and ASMR of CRC attributable to HFPG in 1990 and 2021 and the temporal trends from 1990 to 2021.

Location	Deaths cases (95%UI)		ASMR per 100,000 (95%UI)		AAPC of ASMR (95% CI) 1990–2021
	1990	2021	1990	2021	
Global	31,907 (16,053–48,058)	82,421 (42,427–12,5402)	0.89 (0.45–1.34)	0.98 (0.51–1.49)	0.31 (0.19 – 0.43)
Sex					
Male	16,566 (8,265 – 24,875)	46,559 (23,794 – 71,087)	1.07 (0.54 – 1.61)	1.25 (0.64 – 1.9)	0.51 (0.35 – 0.67)
Female	15,341 (7,726 – 23,104)	35,863 (18,313 – 54,402)	0.76 (0.38 – 1.15)	0.77 (0.39 – 1.16)	0.03 (-0.11 – 0.17)
SDI					
High SDI	15,050 (7,739 – 22,450)	30,579 (15,404 – 46,082)	1.33 (0.69 – 1.99)	1.33 (0.67 – 1.99)	-0.01 (-0.07 – 0.06)
High–middle SDI	9,033 (4,496–13,805)	23,255 (12,027 – 35,689)	0.97 (0.49 – 1.48)	1.17 (0.61 – 1.8)	0.65 (0.46 – 0.84)
Middle SDI	5,479 (2,666 – 8,462)	19,635 (10,122 – 30,542)	0.61 (0.29 – 0.93)	0.77 (0.4 – 1.2)	0.84 (0.61 – 1.07)
Low–middle SDI	1,638 (827 – 2,508)	6,938 (3,441 – 10,555)	0.3 (0.15 – 0.46)	0.52 (0.26 – 0.79)	1.82 (1.71 – 1.93)
Low SDI	657 (320 – 1,024)	1,889 (911 – 2,924)	0.34 (0.16 – 0.53)	0.44 (0.22 – 0.69)	0.92 (0.78 – 1.05)
Region					
Andean Latin America	73 (37 – 114)	435 (215 – 690)	0.39 (0.2 – 0.61)	0.77 (0.38 – 1.21)	2.16 (1.74 – 2.57)
Australasia	334 (166–502)	681 (354–991)	1.42 (0.71–2.13)	1.18 (0.61–1.72)	-0.63 (-0.79 – -0.46)
Caribbean	260 (129 – 395)	746 (370 – 1,172)	1.06 (0.53 – 1.61)	1.38 (0.68 – 2.17)	0.9 (0.8 – 1.01)
Central Asia	165 (81 – 251)	403 (196 – 623)	0.37 (0.18 – 0.55)	0.54 (0.26 – 0.83)	1.35 (0.94 – 1.77)
Central Europe	2,067 (1,052 – 3,061)	5,117 (2,627 – 7,697)	1.42 (0.72 – 2.1)	2.16 (1.11 – 3.25)	1.38 (1.3 – 1.46)
Central Latin America	418 (209 – 631)	2,117 (1,084 – 3,247)	0.57 (0.28 – 0.85)	0.87 (0.44 – 1.34)	1.39 (1.22 – 1.56)
Central Sub–Saharan Africa	88 (45 – 140)	263 (126 – 430)	0.48 (0.24 – 0.75)	0.58 (0.28 – 0.96)	0.62 (0.55 – 0.69)
East Asia	6,527 (3,219 – 9,997)	19,468 (9,807 – 30,265)	0.87 (0.43 – 1.33)	0.92 (0.47 – 1.44)	0.3 (0.01 – 0.59)
Eastern Europe	1,888 (947 – 2,885)	3,869 (1,974 – 5,849)	0.68 (0.34 – 1.04)	1.06 (0.54 – 1.61)	1.45 (0.88 – 2.02)
Eastern Sub–Saharan Africa	251 (119 – 405)	703 (337 – 1,109)	0.41 (0.2 – 0.67)	0.54 (0.26 – 0.85)	0.9 (0.8 – 0.99)
High–income Asia Pacific	2,674 (1384 – 3951)	7,308 (3,687 – 10,994)	1.4 (0.73 – 2.06)	1.29 (0.65 – 1.93)	-0.32 (-0.62 – -0.01)
High–income North America	5,105 (2,653 – 7,625)	9,626 (4,911 – 14,504)	1.4 (0.73 – 2.09)	1.41 (0.72 – 2.12)	-0.01 (-0.17 – 0.16)
North Africa and Middle East	770 (392 – 1,186)	3,626 (1,776 – 5,587)	0.53 (0.27 – 0.82)	0.91 (0.45 – 1.41)	1.78 (1.7 – 1.86)
Oceania	13 (7 – 20)	37 (19 – 58)	0.55 (0.29 – 0.86)	0.59 (0.3 – 0.94)	0.23 (0.12 – 0.34)
South Asia	1,355 (648 – 2,067)	5,426 (2,718 – 8,182)	0.26 (0.12 – 0.4)	0.39 (0.19 – 0.59)	1.41 (1.12 – 1.71)
Southeast Asia	1,110 (542 – 1,735)	5,368 (2,706 – 8,656)	0.51 (0.25 – 0.81)	0.92 (0.46 – 1.49)	1.92 (1.77 – 2.07)
Southern Latin America	492 (245–752)	1,402 (711–2,172)	1.1 (0.55–1.68)	1.56 (0.79–2.41)	1.16 (0.87 – 1.46)
Southern Sub–Saharan Africa	107 (53 – 167)	428 (203 – 663)	0.47 (0.23 – 0.73)	0.86 (0.4 – 1.33)	1.95 (1.62 – 2.27)
Tropical Latin America	494 (245 – 755)	2,390 (1,229 – 3,546)	0.61 (0.3 – 0.94)	0.95 (0.49 – 1.4)	1.45 (1.29 – 1.61)
Western Europe	7,550 (3,791 – 11,405)	12,373 (6,101 – 19,098)	1.23 (0.62 – 1.86)	1.15 (0.57 – 1.77)	-0.22 (-0.32 – -0.12)
Western Sub–Saharan Africa	168 (83 – 264)	635 (294 – 1,002)	0.23 (0.11 – 0.36)	0.4 (0.19 – 0.64)	1.86 (1.79 – 1.93)

**Table 2:** DALYs and ASDR of CRC attributable to HFPG in 1990 and 2021 and the temporal trends from 1990 to 2021.

Location	DALYs cases (95%UI)		ASDR per 100,000 (95%UI)		AAPC of ASDR (95% CI) 1990–2021
	1990	2021	1990	2021	
Global	715,716 (358,249 – 1,089,712)	1,750,923 (900,573–2,657,995)	18.46 (9.27–28.03)	20.31 (10.46–30.81)	0.31 (0.19–0.43)
Sex					
Male	389,682 (194,056 – 592,088)	1,029,880 (520,673 – 1,573,850)	22.12 (11.05 – 33.38)	25.76 (13.05 – 39.44)	0.5 (0.4 – 0.6)
Female	326,034 (162,872 – 492,327)	721,043 (368,503 – 109,4285)	15.49 (7.76 – 23.35)	15.59 (7.97 – 23.67)	0.02 (-0.1 – 0.14)
SDI					
High SDI	308,579 (156,944 – 463,035)	577,527 (295,230 – 861,475)	27.71 (14.1 – 41.57)	27.65 (14.2 – 41.32)	-0.03 (-0.15 – 0.09)
High–middle SDI	207,544 (103,765 – 318,802)	494,756 (252,815 – 766,069)	20.94 (10.47 – 32.11)	24.87 (12.71 – 38.46)	0.57 (0.38 – 0.77)
Middle SDI	139,272 (68,101 – 216,722)	459,950 (235,527 – 714,076)	13.39 (6.53 – 20.72)	16.92 (8.68 – 26.32)	0.81 (0.58 – 1.03)
Low–middle SDI	42,473 (21,671 – 65,607)	170,488 (84,721 – 258,664)	6.86 (3.48 – 10.53)	11.71 (5.82 – 17.78)	1.77 (1.7 – 1.83)
Low SDI	16,746 (8,238–26,052)	45,699 (22,154–70,621)	7.46 (3.65–11.66)	9.27 (4.47–14.31)	0.72 (0.61 – 0.83)
Region					
Andean Latin America	1,571 (793 – 2,487)	451,052 (222,989 – 702,618)	7.96 (4.03 – 12.55)	15.38 (7.52 – 24.39)	2.12 (1.69 – 2.56)
Australasia	7,249 (3,632 – 10,946)	200,781 (103,845 – 300,344)	30.84 (15.43 – 46.5)	24.9 (12.66 – 37.04)	-0.74 (-0.89 – -0.58)
Caribbean	5,460 (2,747 – 8,330)	123,840 (62,644 – 198,168)	21.32 (10.74 – 32.48)	28.77 (14.44 – 45.74)	1 (0.85 – 1.16)
Central Asia	4,239 (2,078–6,448)	9,936 (4,836–15,266)	8.87 (4.36–13.53)	12.06 (5.85–18.57)	1.08 (0.58 – 1.57)
Central Europe	44997 (22687–66970)	82734 (42262–124535)	29.72 (15.04–44.26)	44.68 (22.83–66.76)	1.33 (1.26 – 1.41)
Central Latin America	9,494 (4,767 – 14,211)	979 (506 – 1,542)	11.66 (5.84 – 17.51)	19.66 (10.17 – 29.88)	1.7 (1.59 – 1.82)
Central Sub–Saharan Africa	2,336 (1,182 – 3,704)	101,124 (51,636 – 151,350)	10.55 (5.33 – 16.74)	12.72 (6.1 – 20.82)	0.61 (0.53 – 0.69)
East Asia	169,695 (82,740 – 262,486)	122,009 (62,513 – 182,120)	19.33 (9.54 – 29.8)	20.56 (10.15 – 31.98)	0.27 (-0.04 – 0.59)
Eastern Europe	44,802 (22,524 – 68,640)	216,393 (106,980 – 331,728)	15.72 (7.92 – 24.13)	23.09 (11.8 – 34.79)	1.34 (0.72 – 1.97)
Eastern Sub–Saharan Africa	6,077 (2,910 – 9,696)	27,930 (14,222 – 43,037)	8.59 (4.1 – 13.81)	10.34 (4.98 – 16.3)	0.6 (0.52 – 0.68)
High–income Asia Pacific	58,362 (30,330 – 85,989)	49,644 (25,722 – 75,378)	29.07 (15.14 – 42.8)	25.57 (12.97 – 38.3)	-0.46 (-0.72 – -0.19)
High–income North America	106,584 (54,673 – 157,151)	15,517 (7,784 – 24,658)	30.31 (15.58 – 44.69)	31.31 (16.16 – 46.55)	0.11 (-0.2 – 0.42)
North Africa and Middle East	19,080 (9,610 – 29,426)	8,971 (4,378 – 14,236)	11.45 (5.82 – 17.66)	19.34 (9.52 – 29.77)	1.71 (1.64 – 1.78)
Oceania	348 (181 – 552)	87,065 (42,713 – 133,798)	11.94 (6.22 – 18.73)	13 (6.65 – 20.41)	0.27 (0.14 – 0.4)
South Asia	36,978 (17,750 – 56,172)	137,712 (69,392 – 207,550)	6.16 (2.95 – 9.35)	9.07 (4.57 – 13.65)	1.29 (1.15 – 1.43)
Southeast Asia	26,680 (13,133 – 41,547)	6,947 (3,329 – 11,311)	10.78 (5.28 – 16.85)	19.16 (9.69 – 30.7)	1.89 (1.7 – 2.08)
Southern Latin America	10,310 (5,193 – 15,726)	13,217 (6,762 – 19,457)	22.27 (11.19 – 34.04)	31.83 (16.18 – 48.88)	1.19 (0.92 – 1.46)
Southern Sub–Saharan Africa	2,274 (1,126 – 3,521)	15,724 (7,534 – 24,539)	8.93 (4.41 – 13.82)	17.44 (8.34 – 26.87)	2.18 (1.82 – 2.54)
Tropical Latin America	11,465 (5,702 – 17,469)	9,786 (4,681 – 15,062)	12.78 (6.36 – 19.58)	21.25 (10.86 – 31.78)	1.65 (1.47 – 1.83)
Western Europe	143,915 (72,575 – 216,942)	55,135 (28,144 – 82,455)	24.36 (12.34 – 36.76)	22.7 (11.26 – 34.71)	-0.25 (-0.38 – -0.11)
Western Sub–Saharan Africa	3,797 (1,883 – 5,978)	14,428 (6,672 – 22,827)	4.56 (2.27 – 7.18)	7.92 (3.67 – 12.51)	1.79 (1.68 – 1.9)

Analysis by SDI region level revealed that high SDI region bore the greatest burden in 2021, with 30,579 deaths and 577,527 DALYs. The corresponding ASMR (1.33 per 100,000 population) and ASDR (27.65 per 100,000 population) in the high SDI region were also the highest, being 3.02 times and 2.98 times higher, respectively, than those in the low SDI region (ASMR: 0.44 per 100,000 population, ASDR: 9.27 per 100,000 population), which carry the lowest burden (**Figure 1A**, **Tables 1**, **2**).

Among 21 GBD regions, in 2021, Central Europe had the highest burden, with an ASMR of 2.16 per 100,000 population and ASDR of 44.68 per 100,000 population, followed by Southern Latin America and High-income North America. The highest ASMR in Central Europe (2.16 per 100,000 population) was 5.54 times higher than the lowest ASMR in South Asia (0.39 per 100,000 population), and the highest ASDR in Central Europe (44.68 per 100,000 population) was 5.64 times higher than the lowest ASDR in Western Sub-Saharan Africa (7.92 per 100,000 population) (**Figure 1A**, **Tables 1**, **2**).

At the country level, China reported the highest absolute burden with 18,440 deaths and 429,386 DALYs attributed to HFPG-related CRC in 2021. Regarding ASMR, Barbados (2.62 per 100,000 population), Hungary (2.47 per 100,000 population) and Poland (2.45 per 100,000 population) ranked highest. For ASDR, Hungary (53.69 per 100,000 population), Barbados (52.17 per 100,000 population) and Bulgaria (50.61 per 100,000 population) accounted for the highest rates among 204 countries and territories. Conversely, the lowest ASMR and ASDR were observed in Mozambique, Malawi and Gambia (**Figures 1B, C**, **Supplementary Tables S1**, **S2**).

### 3.2 Temporal trends of colorectal cancer burden attributable to HFPG from 1990 to 2021

**Figure 2:**
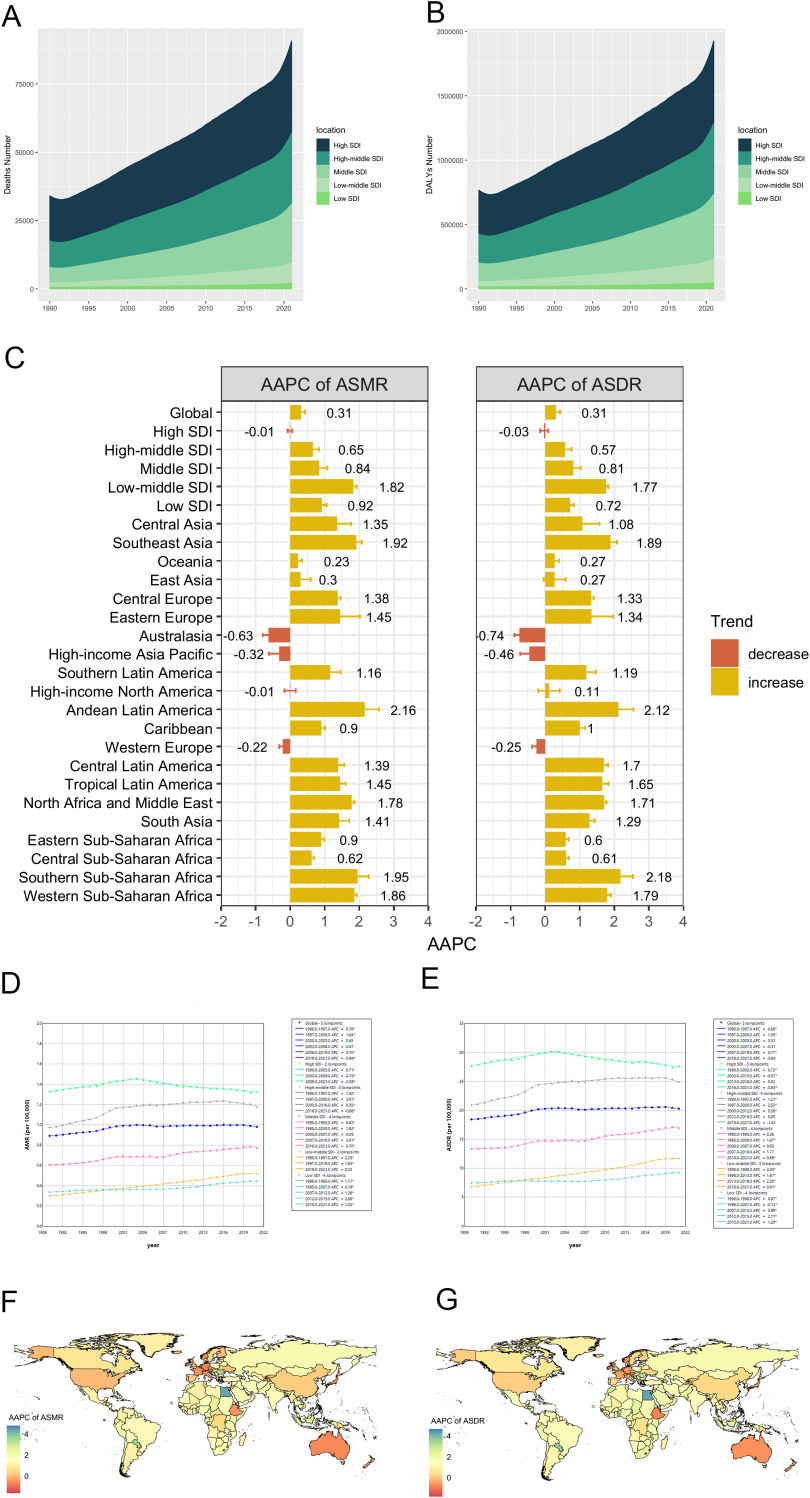
Temporal trends of CRC burden attributable to HFPG from 1990 to 2021. **(A, B)** Deaths and DALYs number from 1990 to 2021 in 5 SDI regions; **(C)** AAPCs of ASMR and ASDR between 1990 and 2021, in global, 5 SDI regions and 21 GBD regions; **(D, E)** Trends of ASMR and ASDR in global and 5 SDI regions, from 1990 to 2021; **(F, G)** AAPC of ASMR and ASDR in 204 countries and territories from 1990 to 2021. DALY, disability-adjusted life-year; SDI, socio-demographic index; ASMR, age-standardized mortality rate; ASDR, age-standardized disability-adjusted life-year rate; AAPC, average annual percentage change.

Over the past three decades, there was a continuous increase in both HFPG-related CRC death and DALYs number on a global scale and within different regions. (**Tables 1**, **2**, **Figures 2A, B**). However, significant variations were observed in ASMR and ASDR. Between 1990 and 2021, the global ASMR and ASDR both exhibited an overall increasing trend, with an AAPC of 0.31 (**Tables 1**, **2**). Notably, while the ASMR showed a steady increase from 1990 to 2019, it subsequently experienced a significant decline through 2021 (APC= -0.94, *p* < 0.05) (**Figure 2D**). The Joinpoint regression analysis also revealed a downward trend in ASDR beginning in 2019, with an APC of -0.69; however, this decline was not statistically significant (*p* > 0.05) (**Figure 2E**).

Among the 5 SDI regions, the high SDI region bore the heaviest disease burden, yet maintained relatively stable ASMR and ASDR from 1990 to 2021 [AAPC: ASMR: -0.01 (95%CI: -0.07–0.06); ASDR= -0.03 (95%CI: -0.15–0.09)] (**Tables 1**, **2**, **Figures 2A–C**). In this region, temporal analysis revealed distinct phases: ASMR demonstrated a steady increase from 1990 to 2003 (APC = 0.71, *p* < 0.05), followed by two periods of decline (2003–2009: APC = -0.79, *p* < 0.05; 2009–2021: APC = -0.38, *p* < 0.05). A similar trend was observed for ASDR, with an inflection point in 2002, marking a subsequent slow decline with different APC, and the most significant decrease occurred between 2016 and 2021 (APC = 0.63, *p* < 0.05). In the high-middle SDI region, ASMR and ASDR followed an overall increasing trend from 1990 to 2021, with patterns resembling those in the high SDI region. Specifically, ASMR showed a significant decline from 2016 to 2021 (APC = -0.56, *p* < 0.05), while ASDR demonstrated a non-significant decline during 2019–2021 (APC = -1.43, *p* > 0.05) (**Figures 2D, E**). In contrast, excluding high and high-middle SDI regions, the other three SDI regions exhibited consistent increases in both ASMR and ASDR over the study period, with the low-middle SDI region showing the most rapid increase [AAPC: ASMR= 1.82 (95%CI: 1.71–1.93); ASDR= 1.77 (95%CI: 1.7–1.83)] (**Tables 1**, **2**, **Figures 2C–E**).

As for 21 regions, significant decreases in both ASMR and ASDR from 1990 to 2021 were observed in Australasia [AAPC: ASMR: -0.63 (95%CI: -0.79– -0.46); ASDR: -0.74 (95%CI: -0.8– -0.58)], High-income Asia Pacific [ASMR: -0.32 (95%CI: -0.62– -0.01); ASDR: -0.46 (95%CI: -0.72– -0.19)], and Western Europe [ASMR: -0.22 (-0.32– -0.12); ASDR: -0.25 (95%CI: -0.38– -0.11)]. Conversely, Andean Latin America experienced the most significant increases in both metrics [AAPC: ASMR: 2.16 (95%CI: 1.74–2.57); ASDR: 2.12 (95%CI: 1.69–2.56)] (**Tables 1**, **2**, **Figure 2C**).

At country level, from 1990 to 2021, Cabo Verde demonstrated the most substantial increases in ASMR and ASDR between 1990 and 2021, with AAPCs of 4.75 (95%CI: 3.94–5.55), while Singapore showed the most significant decreases, with AAPCs of -1.62 (95%CI: -2.02– -1.22) and -1.81 (95%CI: -2.19– -1.42) (**Supplementary Tables S1**, **S2**, **Figures 2E, F**).

### 3.3 Global burden of colorectal cancer burden attributable to HFPG by sex and age

**Figure 3:**
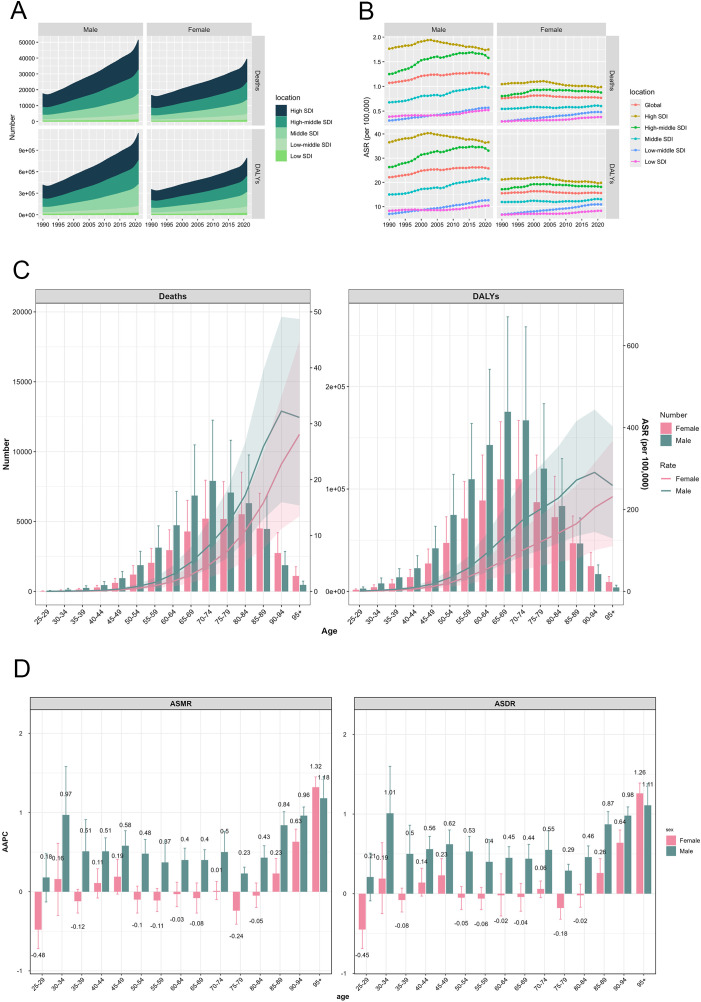
CRC burden attributable to HFPG by sex and age. **(A)** Trends of deaths and DALYs number in 5 SDI regions, by sex, from 1990 to 2021; **(B)** Trends of ASMR and ASDR in 5 SDI regions, by sex, from 1990 to 2021; **(C)** CRC attributable to HFPG by sex and age in 2021; **(D)** AAPC of ASMR and ASDR by sex and age, from 1990 to 2021. DALYs, disability-adjusted life-years; SDI, socio-demographic index; ASMR, age-standardized mortality rate; ASDR, age-standardized disability-adjusted life-year rate; AAPC, average annual percentage change.

From 1990 to 2021, the global burden of CRC attributable to HFPG was consistently greater in males than in females. During this period, male deaths increased substantially from 16,566 to 46,559, while female deaths rose from 15,341 to 35,863. Concurrently, the male-to-female mortality ratio demonstrated an upward trend, increasing from 1.08 to 1.29 (**Tables 1**, **2**, **Figure 3A**). This sex-disparity was further reflected in the ASMR (**Figure 3B**). Specifically, the ASMR for males demonstrated a significant upward trajectory, increasing from 1.07 to 1.25, with an AAPC of 0.51 (95% CI: 0.35–0.67). In contrast, the ASMR for females remained stable, with a negligible AAPC of 0.02 (95% CI: -0.11–0.17). Across 5 SDI regions, males consistently presented higher deaths number and ASMR than females over the 32-year period, and the high SDI region maintain prominence in both genders. Similar sex-specific patterns were observed in DALYs-related indicators globally and across SDI regions. (**Tables 1**, **2**, **Figures 3A, B**).

The age-specific distribution of HFPG-attributable colorectal cancer burden in 2021 revealed similar patterns between genders. I The mortality numbers followed an initial increase followed by a decline, with males reaching peak mortality at ages 70–74 years and females at 80–84 years. The age-specific DALYs number demonstrated comparable patterns, though peaking earlier at 65–69 years in males and 70–74 years in females (**Figure 3C**). Males exhibited higher death and DALYs numbers across all age groups until 85 years, after which females showed higher values (**Figure 3C**). Difference from absolute number of death and DALYs, both ASMR and ASDR were consistently higher in males, but their age-related trajectories differed between genders. Females displayed a steady increase in both ASMR and ASDR with age, whereas males showed an initial rise followed by a decline, reaching the apex within the 90–94 age group (**Figure 3C**).

Temporal analysis across age groups from 1990 to 2021 revealed significant age-specific trends in mortality and disability rates. In males, both ASMR and ASDR across age groups above 30 years showed increasing trends with significant positive AAPCs (*p* < 0.05). The most pronounced increases observed in the over 95 years group, with AAPC of 1.18 (95%CI: 0.91–1.45) for ASMR and 1.11 (95%CI: 0.84–1.38) for ASDR. Female patterns were more variable, with ASMR increasing in age groups over 85 years, and ASDR rising in the 45–49 years and over 85 years age groups. Conversely, both ASMR and ASDR decreased in the 25–29 years and 75–79 years age groups. The age groups in female exhibiting the most pronounced increases and decreases in ASMR and ASDR were congruent, with peak changes occurring in the 90–95 years and 25–29 years age group, respectively (**Figure 3D**).

### 3.4 The relationship between colorectal cancer burden attributable to HFPG and SDI

**Figure 4:**
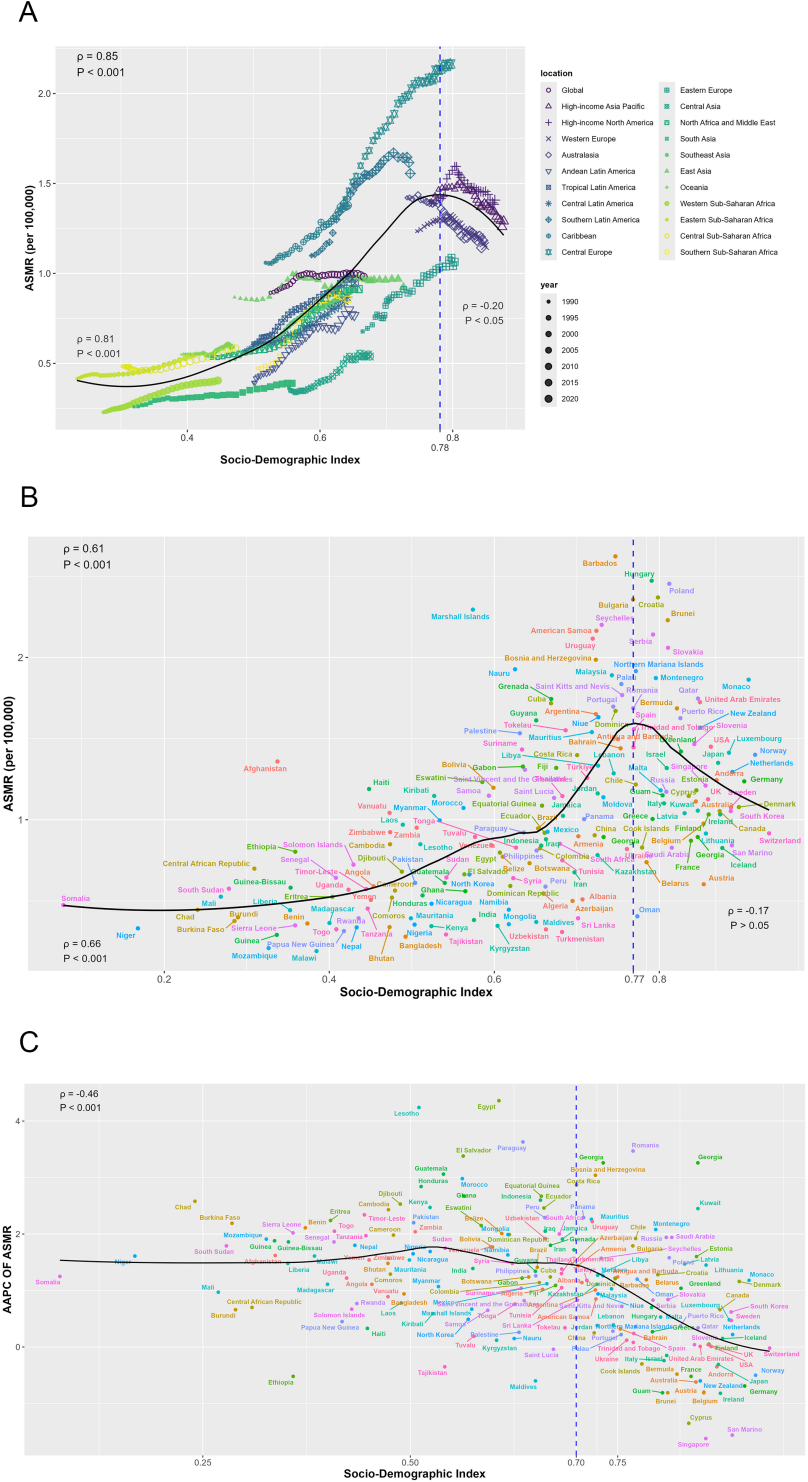
The relationship between mortality of CRC burden attributable to HFPG and SDI. **(A)** The relationship between ASMR and SDI by regions; **(B)** The relationship between ASMR and SDI by countries in 2021; **(C)** The relationship between AAPC of ASMR and SDI by in 2021. ASMR, age-standardized mortality rate; ASDR, age-standardized disability-adjusted life-year rate; AAPC, average annual percentage change.

**Figure 4A** presents a comprehensive analysis of the non-linear association between the ASMR across a global perspective and within 21 regions. A notable inflection point is observed at an SDI value of approximately 0.78, which demarcates two distinct correlation patterns. In the pre-inflection phase, an extremely strong positive correlation was established between SDI and ASMR (ρ = 0.81, *p* < 0.001), indicating that ASMR increases proportionally with rising SDI values. Conversely, post-inflection, the relationship shifts to a weak negative correlation (ρ = -0.20, *p* < 0.05) as SDI continues to increase.

At the country level in 2021, ASMR and SDI exhibit an inverted V-shaped relationship, peaking at an SDI value of approximately 0.77. Before this peak, a strong positive correlation is observed (ρ = 0.66, *p* < 0.001) (**Figure 4B**). Additionally, the AAPC of ASMR exhibited a significant moderate negative correlation with SDI values in 2021 (ρ = -0.46, *p* < 0.001).

Similar patterns were observed for ASDR: the relationship between ASDR and SDI at both global and regional levels, as well as between country-level ASDR and SDI in 2021, mirrored that of ASMR. Specifically, the AAPC of ASDR from 1990 to 2021 also exhibits a comparable correlation with 2021 SDI values (**Supplementary Figure S1**).

### 3.5 Prediction of colorectal cancer burden attributable to HFPG, from 2022 to 2040

**Figure 5:**
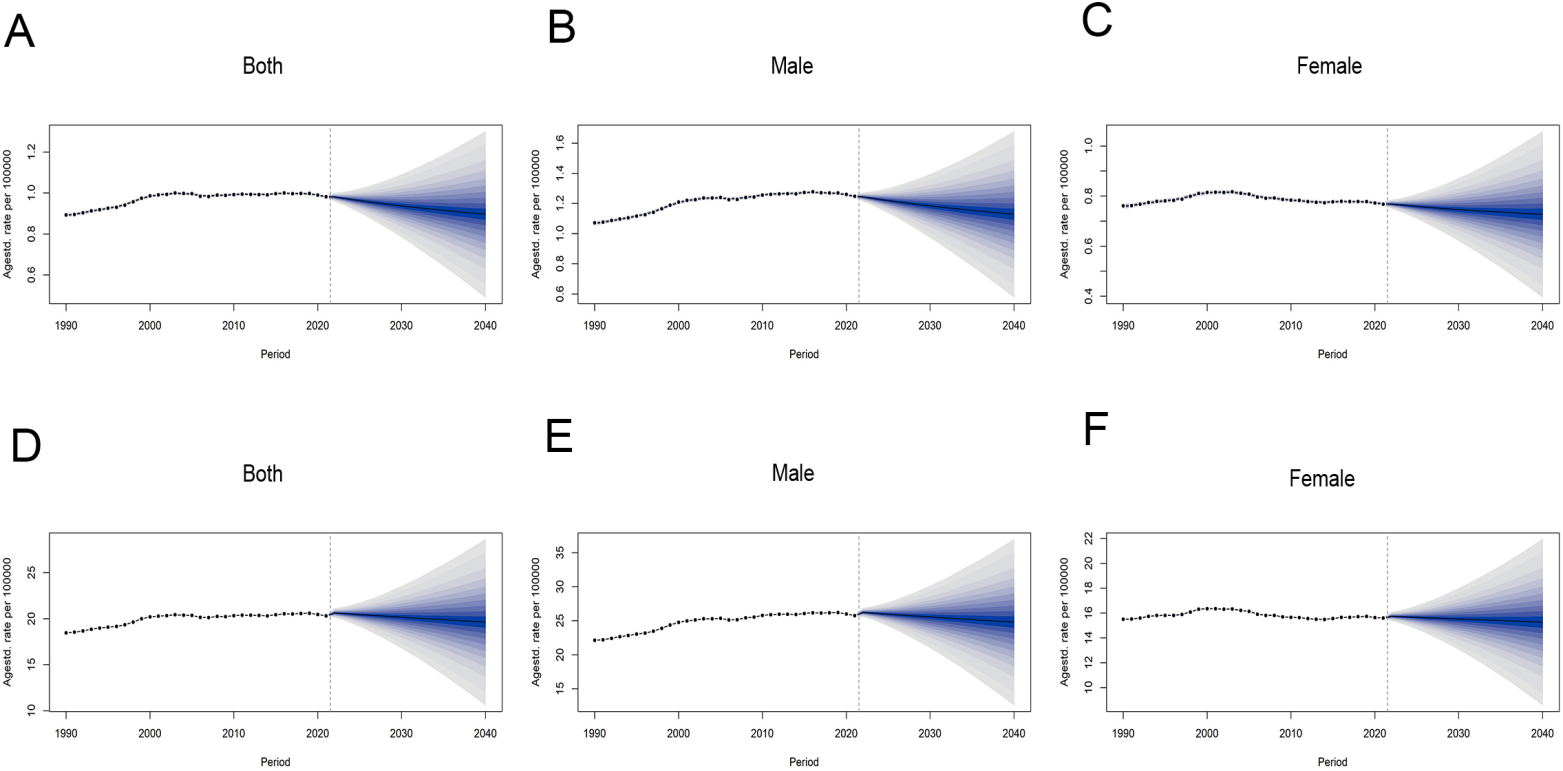
Global trends in ASRs of CRC attributable to HFPG by sex, from 1990 to 2040. **(A–C)** ASMR for all sexes, males and females. **(D–F)** ASDR for all sexes, males and females. ASRs, age-standardized rates; ASMR, age-standardized mortality rate; ASDR, age-standardized disability-adjusted life-year rate.

By 2040, ASMR for CRC attributable to HFPG is projected to decline globally, with a more pronounced decrease in male than in females. For males, the ASMR is anticipated to decrease by 9.56%, from 1.25 per 100,000 population in 2021 to 1.13 per 100,000 population in 2040. In females, the projected decline is 5.44%, from 0.77 per 100,000 population in 2021 to 0.73 per 100,000 population in 2040 (**Figure 5A–C**, **Supplementary Table S3**). As for ASDR, prediction based on BAPC model shows a slight downward trend in both sexes from 2022 to 2040, with changes not exceeding 4% (**Figures 5D–F**, **Supplementary Table S3**).

## 4 Discussion

Colorectal cancer (CRC), as one of the leading cancers worldwide, presents a significant public health challenge due to its substantial disease burden. Recent epidemiological evidence has established a compelling association between hyperglycemia and diabetes and elevated risks of CRC incidence and mortality (24–26). HFPG, a modifiable risk factor, has emerged as a crucial contributor to CRC’s disease burden. Utilizing the GBD 2021 database, our study found that while CRC mortality and DALYs attributable to HFPG showed a slight overall increase from 1990 to 2021, a notable decline emerged after 2019. Significant regional disparities were observed, with high SDI regions shouldering the heaviest burden but showing gradual improvement, while low SDI regions demonstrated accelerating burden increases. The burden of HFPG-related CRC posed more formidable challenges to male and elderly populations. The predicted results indicated that the disease burden would decrease over the next two decades.

From 1990 to 2021, the number of mortality and DALYs of CRC attributable to HFPG consistently rose globally, with slight increases in corresponding ASRs, suggesting that population growth may be primarily responsible for the increase in absolute deaths and DALYs. However, Joinpoint regression analysis revealed a reversal toward declining ASMR and ASDR from 2019 to 2021. Further analysis across 5 SDI regions showed that this decline was predominantly observed in high SDI and high-middle SDI regions, with high SDI region initiating this trend even approximately a decade earlier. Analysis across 21 regions and 204 countries confirmed that burden reductions were primarily concentrated in economically developed areas, such as Australasia, Western Europe, Singapore, and Germany. This may be attributed to superior public health awareness, advanced healthcare infrastructure and medical technologies, as well as the implementation of early CRC screening in these regions. According to the estimation within Americans, approximately 46–63% of CRC deaths were attributed to missed screening opportunities (27). Current screening methodologies, including fecal immunochemical tests, multi-target stool DNA test and colonoscopy, facilitate early detection of adenomas, serrated polyps and advanced adenomas, thereby reducing the incidence and mortality of CRC to varying degrees, with colonoscopy being the most effective (28). Several observational studies found that colonoscopy was associated with a 31–91% reduction in CRC incidence and a 29–88% reduction in CRC-specific mortality, which were higher than other screening methods (28–31). The varying effectiveness of colonoscopy across studies is partly due to differences in the detection of precancerous lesions and early-stage cancer, which depend heavily on the endoscopist expertise and lesion recognition capabilities (32, 33). In economically developed regions, more rigorous training for endoscopists enhances their ability to identify lesions, thereby significantly lowering CRC incidence and mortality (34). Therefore, for lower SDI regions, it is imperative to learn from the experiences of high SDI areas, enhance the construction of basic health facilities, strengthen the chronic disease management of diabetes, and improve the awareness and proficiency of endoscopists.

Furthermore, the observed global decline of ASMR and ASDR after 2019 may partly correlate with the impact of the COVID-19 pandemic, which began in 2019. The pandemic severely disrupted healthcare systems worldwide, diverting resources and attention toward pandemic management at the expense of routine care for chronic diseases such as diabetes. This shift resulted in poor glycemic control among diabetic patients, leading to increased diabetes-related mortality from cardiovascular complications and ketoacidosis (35–38). Consequently, the disease burden of CRC attributable to HFPG may have been indirectly reduced. This phenomenon is consistent with the concept of “competing mortality” observed in epidemiological studies, where the presence of one disease or factor reduces the likelihood of death from another disease or factor. In this context, the excess mortality directly caused by COVID-19 further amplified the competing mortality effect. Specifically, some individuals who might have otherwise succumbed to CRC progression instead died from COVID-19, thereby lowering the observed CRC mortality rate.

Overall, the disease burden of CRC attributed to HFPG was positively correlated with the SDI value, particularly when the SDI was less than 0.8. These findings underscore the importance of considering socioeconomic factors in CRC prevention strategies to address regional health disparities. However, significant disparities in disease burden still exist among regions and countries with similar SDI levels due to differences in population size, increasing aging, lifestyle, genetic predispositions, and healthcare conditions. As shown in our study, China had the largest number of CRC deaths and DALYs attributed to HFPG among 204 countries in 2021, largely due to its large population. Ethnicity also significantly influences this outcome. Prior study has identified ethnicity as a key etiological factor for CRC in Asia, with Chinese, Korean, and Japanese populations showing higher CRC incidence rates. In multi-ethnic nations such as Singapore and Malaysia, the Chinese population exhibits a notably higher CRC incidence rate than Malays and Indians, even when environmental, lifestyle, and dietary factors are comparable (39). Importantly, the ASMR and ASDR in China were not high, even slightly lower than the global average, and these rates remained relatively stable from 1990 to 2021, contrasting with the upward trends observed in previous study (18). In recent years, with economic development and increased government attention to healthcare, China has progressively strengthened diabetes management and screening efforts for CRC. As early as 2018, Chinese guidelines lowered the recommended age for CRC screening to 40 years, particularly for high-risk populations (40). Diabetes, being one of the significant risk factors for CRC, has been incorporated into the risk assessment scoring criteria in several CRC risk evaluation models in China (41, 42). These initiatives have enhanced the early detection and treatment of HFPG-related CRC in China, thereby helping to mitigate the associated disease burden.

Consistent with previous reported, the overall HFPG-related CRC burden was heavier in males compared to females (18). However, our further research findings indicated a notable shift occurs after age 85, where females exhibit higher numbers of deaths and DALYs, despite maintaining lower ASMR and ASDR. This suggests multiple factors influencing the sex-specific CRC burden. Primarily, the higher prevalence of hyperglycemia and diabetes in males, contributes substantially to their elevated CRC burden. Besides, a cross-sectional study across 55 low-income and middle-income countries reported that superior diabetes treatment coverage than among females, potentially mitigating their HFPG-related CRC risk (43). Additionally, biological factors also play a crucial role. Estrogen level, which is higher in females, may be a critical factor in the observed sex disparity. Patients with diabetes often develop hyperinsulinemia, and studies have indicated that higher estrogen in females can enhance the sensitivity of peripheral tissues to insulin, thereby partially counteracting the pro-carcinogenic effects of hyperinsulinemia (44, 45). Moreover, the “sex hormone-gut microbiome axis” may contribute to this disparity as well. Researches have shown that estrogen can promote the diversity of gut microbiome and reduce pathogen enrichment, decreasing the risk of CRC. Conversely, androgen disrupts intestinal microecology and increases the proportion of opportunistic pathogens, exacerbating colonic inflammation and promoting tumorigenesis (46). Lastly, behavioral factors further contribute to the observed differences. Females typically demonstrate greater health consciousness, particularly regarding blood sugar management, and more actively engage in healthcare seeking and primary prevention (47, 48). This proactive approach to health likely contributes to their lower ASMR and ASDR in all age groups. The phenomenon of females surpassing males in terms of mortality and DALYs numbers in the elderly population may be attributed to the fact that females generally have a longer lifespan than males (49, 50).

Our study also confirmed the positive correlation between CRC burden attributable to HFPG and age, which may be related to the progressive degeneration of physical function. Notably, over the past three decades, the increase in CRC burden among males aged 30–34 has been surpassed only by that observed in individuals aged 95 and above. The specific factors driving this phenomenon remain to be elucidated, though it may be linked to the life stress, unhealthy dietary patterns and adverse lifestyle of this demographic. Future researches are warranted to further explore these potential connections and to identify additional factors that may influence CRC burden in these age groups. Such insights could have important implications for the development of targeted healthcare policies and interventions aimed at mitigating CRC risk in high-burden populations.

In this study, we first utilized the BAPC model to forecast the future trends of CRC burden attributable to HFPG through 2040. Our analysis indicates an overall decline in CRC burden for both sexes over the forthcoming 19 years. This projection suggests that ongoing global healthcare policies and advances in medical interventions may be contributing to effective disease prevention and control. Crucially, the BAPC projections operate on the premise that temporal patterns arise from the interplay of age, period, and cohort effects, extrapolating based on historical trend continuity. While this provides a mathematically robust framework for forecasting, several factors could introduce uncertainty into these projections, including variability in baseline data, potential long-term trend modifications, and possible unidentified cohort effects (51). Notably, in this study, the COVID-19 pandemic may have disrupted historical data patterns, potentially affecting the accuracy of future projections. This highlights the need for cautious interpretation of the model outputs and the continuous refinement of forecasting methodologies to account for unforeseen events and their impacts on disease burden. Consequently, our projections should be interpreted as conditional scenarios based on current epidemiological dynamics rather than deterministic forecasts. Continuous methodological refinement incorporating real-time surveillance data remains essential to enhance predictive robustness within evolving global health contexts.

This study represents the first comprehensive analysis of global cross-country health inequalities in CRC burden attributable to HFPG from 1990 to 2021, utilizing newly published data from the GBD 2021 database. Our study extends previous research by examining the associations between disease burden and sex, age groups, and SDI in detail. Moreover, we employed Joinpoint regression analysis to assess the temporal trends in disease burden and explored the impact of the COVID-19 pandemic on the changes in disease burden. We also developed projections of disease burden trends extending to 2040. However, several limitations warrant consideration. Firstly, the completeness and accuracy of the data remain a concern. Although the GBD database encompasses nearly all available data sources, numerous low-SDI regions and countries still face challenges such as misdiagnosis, missed diagnosis, underreporting, and data gaps, primarily attributable to inadequate healthcare infrastructure and flawed cancer registration systems (10, 52). Secondly, the GBD dataset used in this study covers an extensive temporal and spatial scope, which inevitably introduces discrepancies in measurement standards, potentially interfering with the analysis results (53). Thirdly, the 95% UIs presented in this study do not account for various potential biases, including selection bias, measurement bias, and model specification bias (4). Fourthly, although the CRA framework adjusted for major confounders, residual confounding from unmeasured factors such as genetic interactions or inflammatory markers may affect estimates of CRC burden attributable to HFPG. The resulting burden estimates represent population-level attributable risk derived through GBD’s standardized methodology, reflecting statistical associations rather than direct causal relationships, which constitutes a recognized limitation in secondary analyses of GBD data. Finally, this study cannot explicitly evaluate effect modification by HFPG-related comorbidities, potentially overlooking synergistic impacts on CRC pathogenesis. Overall, these limitations highlight the need for cautious interpretation of the findings, especially when extrapolating the results to regions or populations with different data quality and healthcare contexts. Future research should continue to explore the impact of these limitations on the assessment of CRC burden and strive to improve the accuracy and reliability of disease burden estimates.

## 5 Conclusion

In conclusion, this study demonstrated that from 1990 to 2021, the mortality and DALYs rate of CRC attributed to HFPG showed a slight overall increase However, a decline was observed starting from 2019, which may be associated with improved blood glucose management, expanded screening coverage, and the impact of the COVID-19 pandemic. At regional and national levels, the reduction in the burden of CRC was primarily evident in high SDI or economically developed regions and countries. Moreover, higher ASMR and ASDR were noted among the elderly and male populations. Notably, within the young male population, an alarming upward trend was observed, particularly in the 30–34 age group. According to the projections made in this study, by 2024, both the ASMR and ASDR are expected to show decreasing trends in both male and female populations. These findings provide a theoretical basis for the development of more targeted prevention and early screening strategies to address the disparities observed across different demographic groups and regions.

## Data Availability

The original contributions presented in the study are included in the article/**Supplementary Material**. Further inquiries can be directed to the corresponding author.
