# Supplementary figures and images for: Global burden of colorectal cancer attributable to high fasting plasma glucose from 1990 to 2021 and projection to 2040
Source: Front Oncol. 2025 Aug 26;15:1590382. doi: 10.3389/fonc.2025.1590382 (PMC12418600; doi:10.3389/fonc.2025.1590382)

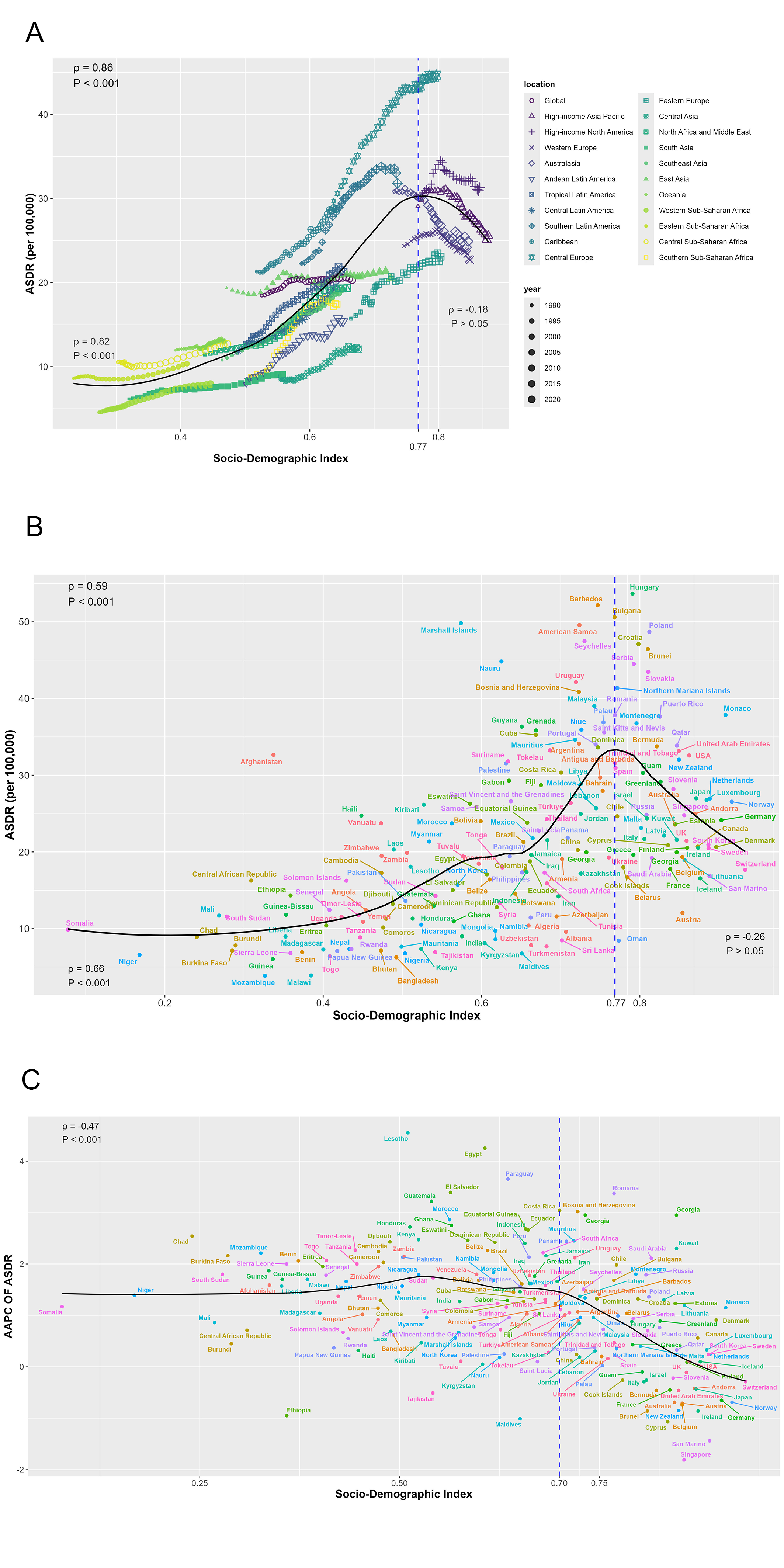

Supplement: Supplementary file 1 [file Image1.tif]
